# Luminal Cerebrovascular Proteomics

**DOI:** 10.21769/BioProtoc.5411

**Published:** 2025-08-05

**Authors:** Sophia M. Shi, Carolyn R. Bertozzi, Tony Wyss-Coray

**Affiliations:** 1 Department of Chemistry, Stanford University, Stanford, CA, USA; 2 Stanford Chemistry, Engineering & Medicine for Human Health (ChEM-H), Stanford University, Stanford, CA, USA; 3 Department of Neurology and Neurological Sciences, Stanford University School of Medicine, Stanford, CA, USA; 4 Wu Tsai Neurosciences Institute, Stanford University School of Medicine, Stanford, CA, USA; 5 Howard Hughes Medical Institute, Stanford University, Stanford, CA, USA; 6 The Phil and Penny Knight Initiative for Brain Resilience, Stanford University, Stanford, CA, USA

**Keywords:** Vasculature, Blood–brain barrier, Luminal, Proteomics, CNS, Brain endothelial cells, Aging, Neurodegeneration

## Abstract

Brain endothelial cells, which constitute the cerebrovasculature, form the first interface between the blood and brain and play essential roles in maintaining central nervous system (CNS) homeostasis. These cells exhibit strong apicobasal polarity, with distinct luminal and abluminal membrane compositions that crucially mediate compartmentalized functions of the vasculature. Existing transcriptomic and proteomic profiling techniques often lack the spatial resolution to discriminate between these membrane compartments, limiting insights into their distinct molecular compositions and functions. To overcome these limitations, we developed an in vivo proteomic strategy to selectively label and enrich luminal cerebrovascular proteins. In this approach, we perfuse a membrane-impermeable biotinylation reagent into the vasculature to covalently tag cell surface proteins exposed on the luminal side. This is followed by microvessel isolation and streptavidin-based enrichment of biotinylated proteins for downstream mass spectrometry analysis. Using this method, we robustly identified over 1,000 luminally localized proteins via standard liquid chromatography–tandem mass spectrometry (LC–MS/MS) techniques, achieving substantially improved enrichment of canonical luminal markers compared with conventional vascular proteomic approaches. Our method enables the generation of a high-confidence, compartment-resolved atlas of the luminal cerebrovascular proteome and offers a scalable platform for investigating endothelial surface biology in both healthy and disease contexts.

Key features

• Enables high-resolution proteomic profiling of the luminal surface of the brain vasculature in vivo.

• Improves signal-to-noise ratio through an added microvessel isolation step, reducing nonspecific background.

• Applied to uncover aging-related changes in luminal endothelial surface protein composition.

• Adaptable for identifying therapeutic targets, transporters, signaling pathways, and disease-associated alterations in the luminal vascular environment across diverse biological contexts.

## Graphical overview


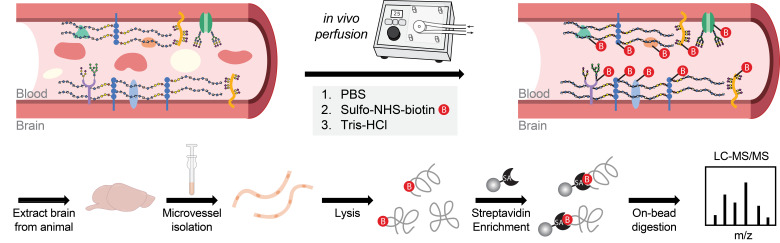


**Luminal cerebrovascular proteomics workflow**

## Background

The cerebrovasculature plays a central role in maintaining brain homeostasis by tightly regulating the exchange of molecules between the blood and brain parenchyma. This function relies on the structural and functional polarization of brain endothelial cells, whose distinct luminal and abluminal surfaces mediate specialized interactions with the circulating milieu and neural microenvironment, respectively [1–3]. The luminal membrane serves as the primary interface with the bloodstream, harboring proteins that sense systemic signaling molecules, modulate vascular permeability, regulate immune surveillance, and support a distinct glycocalyx layer [3–5]. Despite its importance, previous cerebrovascular proteomic studies have often failed to capture the luminal proteome with high specificity, either identifying a limited number of luminal proteins or lacking the spatial resolution to distinguish between the luminal and abluminal compartments [6–8]. To address these limitations, we developed a perfusion-based protocol using a slow, peristaltic pump system to deliver a membrane-impermeable biotinylation reagent that selectively labels surface-exposed proteins on the luminal side of the cerebrovasculature. This is followed by vascular enrichment [9–11] and streptavidin-based protein capture for downstream protein identification via LC/MS–MS [12,13], enabling high-specificity isolation of luminal proteins with reduced background contamination and improved protein recovery. Using this approach, we identified over 1,000 luminal surface proteins from the brain vasculature in mice, the majority of which are annotated as cell surface proteins involved in key biologically relevant pathways such as transport, adhesion, communication, signaling, and extracellular matrix organization. This method offers a robust and scalable platform for in vivo mapping of the luminal cerebrovascular proteome and can be directly used for applications such as revealing biomarkers of vascular dysfunction and discovering new surface receptors for targeted CNS drug delivery. Furthermore, direct comparison of luminal-specific and whole-surface vascular proteomics provides a powerful approach to resolve the molecular distinctions between luminal and abluminal compartments and deepen our understanding of endothelial polarity in vivo.

## Materials and reagents

**Biological materials**

1. C57BL/6J mice (Jackson Laboratory, catalog number: 000664)

**Reagents**

1. Phosphate-buffered saline (PBS) (Thermo Fisher Scientific, catalog number: 10010049)

2. HBSS, calcium, magnesium, no phenol red (Thermo Fisher Scientific, Gibco^TM^, catalog number: 14025092)

3. 2,2,2-tribromoethanol (Sigma-Aldrich, catalog number: T48402)

4. 2-methyl-2-butanol (Sigma-Aldrich, catalog number: 240486)

5. UltraPure^TM^ 1 M Tris-HCl buffer, pH 7.5 (Thermo Fisher Scientific, catalog number: 15567027)

6. Bovine serum albumin (BSA) (Thermo Fisher Scientific, catalog number: 50-253-900)

7. Sulfo-NHS-Biotin (Thermo Fisher Scientific, catalog number: 21217)

8. cOmplete^TM^, EDTA-free protease inhibitor (PI) cocktail (Thermo Fisher Scientific, catalog number: 11873580001)

9. Dextran from *Leuconostoc mesenteroides* (Millipore Sigma, catalog number: D8821)

10. RIPA lysis and extraction buffer (Thermo Fisher Scientific, catalog number: 89901)

11. Pierce^TM^ BCA Protein Assay kit (Thermo Fisher Scientific, catalog number: 23225)

12. Pierce^TM^ streptavidin magnetic beads (Thermo Fisher Scientific, catalog number: 88816)

13. Urea (Millipore Sigma, catalog number: 23225)

14. Iodoacetamide (IAA) (Millipore Sigma, catalog number: I1149)

15. Dithiothreitol (DTT) (Thermo Fisher Scientific, catalog number: R0861)

16. Trypsin/Lys-C Mix, mass spec grade (Promega, catalog number: V5073)

17. Water (LC–MS grade) (Thermo Fisher Scientific, catalog number: 51140)

18. Formic acid (LC–MS grade) (Thermo Fisher Scientific, catalog number: 28905)

19. Acetonitrile (LC–MS grade) (Thermo Fisher Scientific, catalog number: 51101)

20. Methanol (LC–MS grade) (Thermo Fisher Scientific, catalog number: A456)

**Solutions**

1. Avertin (see Recipes)

2. 1% BSA (see Recipes)

3. 1% BSA + 1× PI (see Recipes)

4. 32% Dextran (see Recipes)

5. 0.5 mg/mL Sulfo-NHS-biotin (see Recipes)

6. 50 mM Tris-PBS (see Recipes)

7. RIPA lysis buffer +1× PI (see Recipes)

8. 50mM Tris-HCl (pH 7.5) (see Recipes)

9. 2 M Urea in 50 mM Tris (see Recipes)

10. 2 M Urea in 50 mM Tris with 1 mM dithiothreitol (DTT) and 0.4 μg trypsin/LysC (see Recipes)

11. 0.1% Formic acid in LC–MS-grade water (solvent A) (see Recipes)

12. 0.1% Formic acid in 80% LC–MS-grade acetonitrile (solvent B) (see Recipes)

13. 0.1% Formic acid in LC–MS-grade acetonitrile (solvent Y) (see Recipes)

**Recipes**

**1. Avertin (2.5% v/v)**

Prepare in advance. 40× Avertin stocks are prepared prior by dissolving 10 g of 2,2,2-triboromoethanol in 10 mL of 2-methyl-2-butanol using gentle warming (~50 °C) and stirring in the dark. Store 1 mL aliquots of 40× Avertin at -80 °C for up to 12 months. Prepare 1× Avertin by mixing 1 mL of 40× Avertin with 39 mL of PBS. Store at 2–8 °C protected from light for up to 4 weeks.

**Table:** 

Reagent	Final concentration	Quantity or Volume
Avertin (40× stock)	1× (2.5% v/v)	1 mL
PBS	n/a	39 mL
Total	n/a	40 mL

**2. 1% BSA**

Add 5 g of BSA to a sterile 500 mL bottle of 1× PBS and mix until dissolved. Store at 2–8 °C for up to 4 weeks.

**Table:** 

Reagent	Final concentration	Quantity or Volume
BSA	1% (w/v)	5 g
PBS	n/a	500 mL
Total	n/a	500 mL

**3. 1% BSA + 1× PI**

Prepare and aliquot 25× PI according to the manufacturer’s instructions. Thaw a vial of 25× PI and add 200 μL of 25× PI to 4,800 μL of 1% BSA in PBS. Prepare 5 mL per sample. Store on ice and make fresh.

**Table:** 

Reagent	Final concentration	Quantity or Volume
25× protease inhibitor	1×	200 μL
1% BSA in PBS (Recipe 2)	n/a	4,800 μL
Total	n/a	5,000 μL

**4. 32% Dextran**

Prepare in advance. Add HBSS to 100 g of Dextran from *Leuconostoc mesenteroides* for a final volume of 312.5 mL. Add a clean magnetic stirring rod and stir at room temperature until dissolved. Can be stored at 4 °C for up to 3 months.

**Table:** 

Reagent	Final concentration	Quantity or Volume
Dextran	32% (w/v)	100 g
HBSS	n/a	Fill to 312.5 mL
Total	n/a	312.5 mL

**5. 0.5 mg/mL Sulfo-NHS-biotin**

Add 10 mg of Sulfo-NHS-biotin to 20 mL of PBS. Vortex until dissolved. Prepare 20 mL of solution fresh per animal and use immediately.

**Table:** 

Reagent	Final concentration	Quantity or Volume
Sulfo-NHS-biotin	0.5 mg/mL	10 mg
PBS	n/a	20 mL
Total	n/a	20 mL

**6. 50 mM Tris-PBS**

Add 0.5 mL of 1 M Tris-HCl buffer (pH 7.5) to 9.5 mL of PBS and vortex until dissolved. Prepare 10 mL of solution per animal. Store at room temperature for up to 1 week.

**Table:** 

Reagent	Final concentration	Quantity or Volume
1 M Tris-HCl buffer, pH 7.5	50 mM	0.5 mL
PBS	n/a	9.5 mL
Total	n/a	10 mL

**7. RIPA lysis buffer + 1× PI**

Prepare and aliquot 25× PI according to the manufacturer’s instructions. Thaw a vial of 25× PI and add 8 μL of 25× PI to 192 μL of RIPA lysis buffer. Prepare 200 μL per sample, plus additional volume for sample dilution as needed. Store on ice and make fresh.

**Table:** 

Reagent	Final concentration	Quantity or Volume
25× protease inhibitor	1×	8 μL
RIPA lysis buffer	n/a	192 μL
Total	n/a	200 μL

**8. 50 mM Tris-HCl (pH 7.5)**

Add 35 μL of 1 M Tris-HCl buffer (pH 7.5) to 665 μL of LC–MS-grade water and vortex to mix. Prepare at least 700 μL per sample. Store at room temperature for up to 1 week.

**Table:** 

Reagent	Final concentration	Quantity or Volume
1 M Tris-HCl buffer, pH 7.5	50 mM	35 μL
LC–MS-grade water	n/a	665 μL
Total	n/a	700 μL

**9. 2 M Urea in 50 mM Tris (pH 7.5)**

Add 60.06 mg of urea to 500 μL of LC–MS-grade water and vortex to mix. Prepare fresh 500 μL of solution per sample.

**Table:** 

Reagent	Final concentration	Quantity or Volume
Urea	2 M	60.06 mg
50 mM Tris-HCl	n/a	500 μL
Total	n/a	500 μL

**10. 2 M Urea in 50 mM Tris with 1 mM DTT and 5 μg/mL trypsin/Lys-C**

Prepare 1 M stocks of DTT by dissolving 30.85 g of DTT in 20 mL of sterile water and store aliquoted stocks at -20 °C. Reconstitute trypsin/Lys-C according to manufacturer instructions at 0.2 μg/μL, and store aliquoted stocks at -80 °C. Add 0.08 μL of 1 M DTT and 2 μL of 0.2 μg/μL Trypsin/LysC to 78 μL of 2 M Urea in 50 mM Tris. Prepare 80 μL of solution fresh per sample.

**Table:** 

Reagent	Final concentration	Quantity or Volume
1 M DTT	1 mM	0.08 μL
0.2 μg/μL Trypsin/LysC	5 μg/mL	2 μL
2 M Urea in 50 mM Tris	n/a	78 μL
Total	n/a	80 μL

**11. 0.1% formic acid in LC–MS-grade water (solvent A)**

Add 0.5 mL of formic acid to 499.5 mL of LC–MS-grade water and mix thoroughly. Store at room temperature for up to 6 months.

**Table:** 

Reagent	Final concentration	Quantity or Volume
LC–MS-grade formic acid	0.1% (v/v)	0.5 mL
LC–MS-grade water	n/a	499.5 mL
Total	n/a	500 mL

**12. 0.1% formic acid in 80% LC–MS-grade acetonitrile (solvent B)**

Mix 0.15 μL of formic acid, 29.85 μL of LC–MS-grade water, and 120 μL of LC–MS-grade acetonitrile. Prepare 150 μL per sample. Store at room temperature for up to 1 month.

**Table:** 

Reagent	Final concentration	Quantity or Volume
LC–MS-grade formic acid	0.1% (v/v)	0.15 μL
LC–MS-grade water	n/a	29.85 μL
LC–MS-grade acetonitrile	n/a	120 μL
Total	n/a	150 μL

**13. 0.1% formic acid in LC–MS-grade acetonitrile (solvent Y)**

Add 0.5 mL of formic acid to 499.5 mL of LC–MS-grade acetonitrile and mix thoroughly. Store at room temperature for up to 6 months.

**Table:** 

Reagent	Final concentration	Quantity or Volume
LC–MS-grade formic acid	0.1% (v/v)	0.5 mL
LC–MS-grade acetonitrile	n/a	499.5 mL
Total	n/a	500 mL

**Laboratory supplies**

1. Corning^®^ 50 mL centrifuge tubes (Millipore Sigma, catalog number: CLS4558-300EA)

2. Corning^®^ 15 mL centrifuge tubes (Millipore Sigma, catalog number: CLS430790-500EA)

3. Terumo Surflo Winged Infusion Set 25G × 1/2” (Fisher Scientific, catalog number: 22-289911)

4. 30 mL syringe (Fisher Scientific, catalog number: BD302832)

5. Dissection tools

a. Iris scissors, 10 cm, SuperCut, straight, German (World Precision Instruments, catalog number: 14218-G)

b. Iris forceps, 10 cm, curved, serrated, German (World Precision Instruments, catalog number: 15915-G)

c. Student Halsted-mosquito hemostats (Fine Science Tools, catalog number: 91308-12)

6. Wheaton^®^ 357424 glass 7 mL Tenbroeck Tissue Grinder Set, grinding chamber O.D. × L: 16 × 82 mm (Dounce homogenizer) (catalog number: 357424)

7. VWR^®^ disposable Petri dishes, 60 × 15, mono Petri dishes (VWR International, catalog number: 25384-168)

8. VWR^®^ razor blades (VWR International, catalog number: 55411-050)

9. Corning^®^ cell strainer, pore size 40 μm (Corning, Millipore Sigma, catalog number: CLS431750-50EA)

10. Snap Cap low retention microcentrifuge tubes (Thermo Fisher Scientific, catalog number: 3448PK)

11. Eppendorf^TM^ Protein LoBind^TM^ tubes (Thermo Fisher Scientific, catalog number: 13-698-794)

12. DynaMag^TM^-2 magnet (Thermo Fisher Scientific, catalog number: 12321D)

13. BioPureSPN mini C18 columns (The Nest Group, catalog number: HUM S18V)

## Equipment

1. VWR^®^ variable-speed peristaltic pump (VWR, catalog number: 70730-062)

2. Qsonica sonicator Q125 (Fisher Scientific, catalog number: 15-338-283)

3. Centrifuge 5810/5810 R (Eppendorf, catalog number/model: 022625004)

4. Centrifuge 5425 (Eppendorf, catalog number: 5405000042)

5. Pipettes

6. End-over-end tube rotator

7. Labconco^TM^ CentriVap^TM^ benchtop concentrator (Fisher Scientific, catalog number: 16-108-334)

8. LC–MS/MS (Thermo Fisher Scientific, Q Exactive HF-X coupled with UltiMate 3000 RSLCnano system)

## Software and datasets

1. MaxQuant (Max Planck Institute of Biochemistry, Version 1.6.10.43)

2. Perseus (Max Planck Institute of Biochemistry, Version 2.0.11.0)

## Procedure

**A. Luminal cerebrovascular surface biotinylation and microvessel isolation**

1. Anesthetize the mouse via intraperitoneal injection of 2.5% (v/v) Avertin.

2. Using a peristatic pump set to a flow rate of 2–3 mL/min, perform intracardiac perfusion with the following ice-cold solutions in sequence: 8 mL of PBS (to flush out blood), 20 mL of 0.5 mg/mL Sulfo-NHS-biotin (for luminal biotinylation), and 10 mL of 50 mM Tris-PBS (to quench unreacted biotin).

For intracardiac perfusion, expose the chest cavity and make a small incision in the right atrium to allow fluid drainage. Insert the perfusion needle into the left ventricle, ensuring secure placement without puncturing the ventricular wall. Begin perfusion immediately after needle insertion by activating the pump. Switch the source liquid between each solution as indicated.

**Figure 1.**
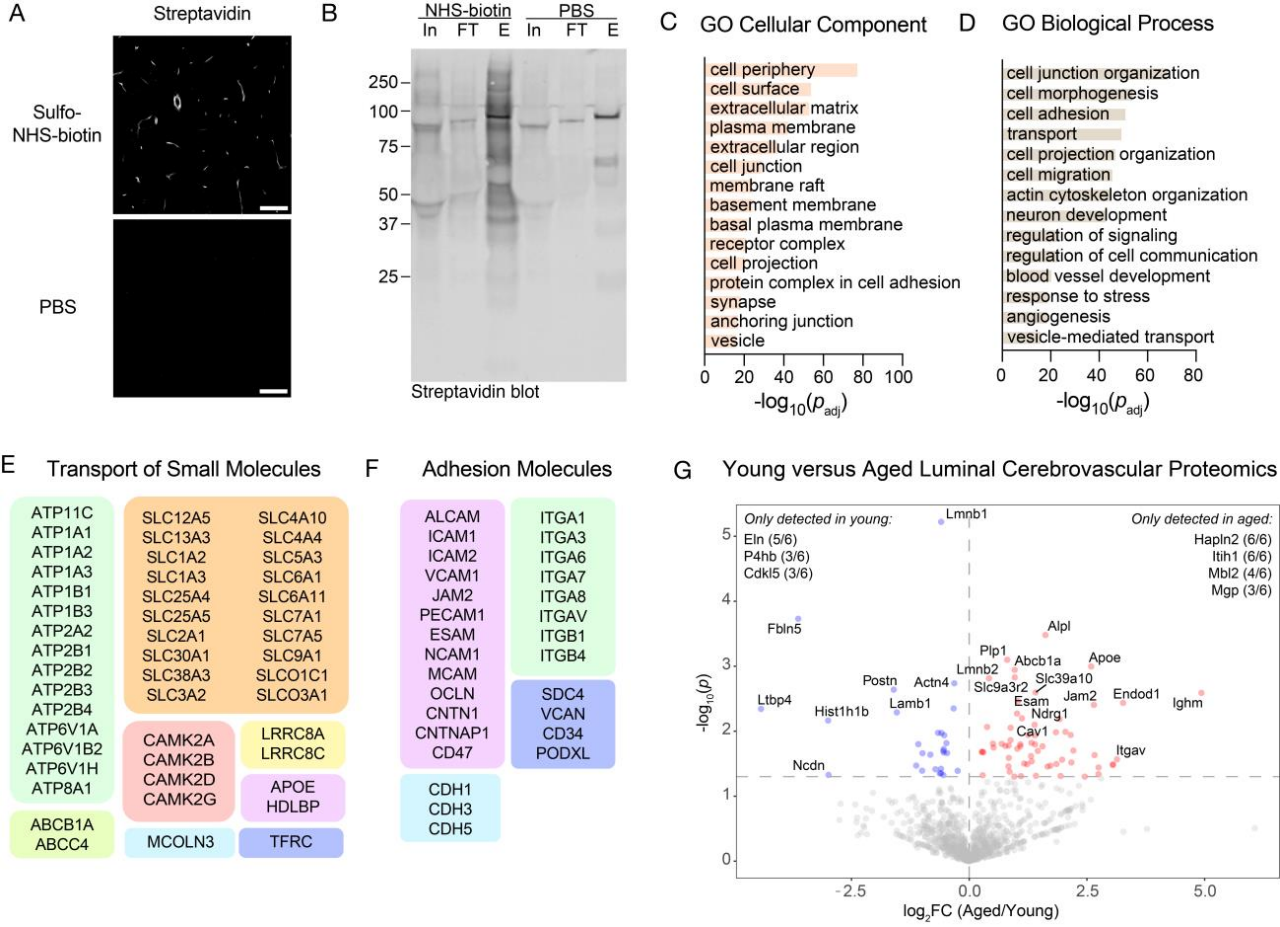
Validation and characterization of luminal cerebrovascular proteomics. (A) Effective cerebrovascular labeling by sulfo-NHS-biotin perfusion via streptavidin detection compared with PBS-perfused mice. Scale bar = 50 μm. (B) Western blot of brain microvessel lysates from mice perfused with PBS or sulfo-NHS-biotin. Biotinylated proteins were enriched using streptavidin beads. In, input; FT, flowthrough; E, eluate. (C) Gene ontology (GO) cellular component analysis of all proteins identified in the luminal cerebrovascular proteomics dataset. (D) GO biological process analysis of all proteins identified in the luminal cerebrovascular proteomics dataset. (E) Representative proteins identified in the luminal proteomics dataset involved in the transport of small molecules. (F) Representative proteins identified in the luminal proteomics dataset involved in cell adhesion. (G) Example application of this procedure to measure luminal cerebrovascular proteins in young (3-month-old) vs. aged (21-month-old) mice (n = 6 mice per group). Volcano plot showing proteins with different abundances identified between the young and aged groups. Peptide and protein identifications were filtered to a 1% false discovery rate (FDR). Adapted from Shi et al. [4], Extended Data Figure 4b–d, 4f.

**Critical:** Ensure effective perfusion with each solution for successful biotinylation of luminal proteins. Cerebrovascular biotinylation can be assessed via streptavidin staining and confocal imaging (Figure 1A).

3. Following intracardiac perfusion, carefully open up the skull using surgical scissors, gently remove the brain using forceps, and store it in cold 1% BSA with 1× protease inhibitor (PI) in PBS on ice.

4. Proceed directly to microvessel isolation.

**Critical:** The microvessel isolation procedure enhances signal-to-noise for luminal vascular protein identification.

*Note: A detailed Bio-Protocol for brain microvessel isolation with procedural images is described in Buff et al. (2025) [11]. Key steps of the protocol are described below.*

5. Place hemibrain in 0.5 mL of 1% BSA + 1× PI in a 60 mm Petri dish on ice.

*Note: If dissecting smaller brain regions, you can scale volumes accordingly.*

6. Mince tissue into ~1 mm^3^ pieces with a razor blade.

7. Transfer the minced brain tissue into a 7 mL Dounce homogenizer on ice using a precut 1,000 μL pipette tip.

8. Carefully wash the blade and dish with 1 mL of 1% BSA + 1× PI and transfer to Dounce homogenizer.

9. Gently dounce the mixture until just homogenized.

**Critical:** There should be no visible chunks of brain tissue left after douncing. This may take ~20 strokes with very little twisting. Keep the Dounce on ice as much as possible to prevent protein degradation.

10. Transfer homogenate to a 15 mL conical tube with 10 mL of chilled 32% dextran.

11. Wash the Dounce homogenizer with 1 mL of 1% BSA + 1× PI and transfer to the same 15 mL conical tube with dextran.

12. Mix tissue homogenate with the 32% dextran by inverting the tube repeatedly until fully homogenized.

13. Centrifuge the dextran-brain lysate mixture at 4,400× *g* at 4 °C for 25 min with slow deceleration for density-based separation of microvessels.

*Note: After centrifugation, three distinct layers should be visible: a white myelin layer (top), a translucent parenchymal cell layer (middle), and a microvessel pellet (bottom).*

14. Remove the myelin layer and parenchymal cell fraction via aspiration, being careful not to disturb the microvessel pellet.

15. Pre-wet the mesh of a 40 μM strainer placed atop a 50 mL conical tube with 5 mL of 1% BSA.

16. Resuspend the pellet in 1 mL of 1% BSA + 1× PI and apply to the pre-wet 40 μM strainer.

17. Gently wash the microvessels with 30 mL of cold PBS through the strainer.

18. Invert the filter on another 50 mL conical tube and collect the microvessels by washing with 20 mL of 1% BSA.

**Critical:** There should be no visible microvessels left on the strainer.

19. Centrifuge the solution at 2,000× *g* for 15 min at 4 °C with slow deceleration.

20. Remove the supernatant as carefully as possible without disturbing the microvessel pellet.

21. Resuspend the pellet in 200 μL of RIPA buffer supplemented with 1× PI and transfer to a 1.5 mL low protein retention microfuge tube.

**Pause point:** Lysates can be stored at -20 °C or -80 °C until future use.

**B. Luminal proteome enrichment and peptide preparation**

1. Lyse microvessels via sonication in RIPA lysis buffer + 1× PI on ice.

*Note: Qsonica recommended settings: 50% amplitude, 10 s on/30 s off, 3 times.*

2. Centrifuge lysates at 13,000× *g* for 15 min at 4 °C and save the supernatant for downstream processing.

3. Measure protein concentration of the supernatant by BCA assay.

4. Incubate 1 mg of each sample diluted to 300 μL of total volume in RIPA lysis buffer + 1× PI with 70 μL of streptavidin magnetic beads at 4 °C overnight with end-over-end rotation.

*Note: Successful enrichment of biotinylated proteins can be evaluated using a streptavidin western blot using input, flowthrough, and elution samples (Figure 1B). Refer to [12] for additional details.*

5. The following day, wash beads at room temperature one time with 200 μL of 50 mM Tris-HCl (pH 7.5) and two times rapidly with 200 μL of 2 M urea in 50 mM Tris (pH 7.5) using a magnetic bead rack for bead-solution separation.

6. Incubate beads with 80 μL of 2 M urea in 50 mM Tris with 1 mM DTT and 0.4 μg of trypsin/Lys-C at 25 °C for 1 h with shaking at 300 rpm.

7. Transfer supernatant to a new low protein retention microfuge tube.

8. Wash beads two times with 60 μL of 2 M urea in 50 mM Tris and combine washes with on-bead digest supernatant.

9. Reduce solution with 4 mM DTT for 30 min with shaking at 300 rpm. For 200 μL of solution, add 4.1 μL of 200 mM DTT to achieve a final concentration of 4 mM DTT.

10. Alkylate solution with 10 mM iodoacetamide (IAA) for 45 min in the dark with shaking at 300 rpm. For 204.1 μL of solution, add 5.2 μL of 400 mM IAA to achieve a final concentration of 10 mM IAA.

11. Add an additional 0.5 μg of trypsin/Lys-C (2.5 μL of 0.2 μg/μL trypsin/Lys-C stock) to each sample for overnight digestion at 37°C with shaking.

12. The following day, acidify samples to 1% (v/v) formic acid. For 212 μL of solution, add 2.1 μL of formic acid to achieve a final concentration of 1% (v/v) formic acid.

13. Desalt peptide solution using C18 columns according to the manufacturer’s instructions. All centrifuge steps are carried out at 50× *g* for 1 min at room temperature. In brief:

a. Wash columns with 200 μL of methanol and equilibrate two times with 200 μL of 0.1% formic acid in LC–MS-grade water (solvent A).

b. Load samples onto columns and centrifuge.

c. Wash columns four times with 200 μL of solvent A via centrifugation.

d. Elute peptides from the columns two times into new low protein retention microfuge tubes with 75 μL of 0.1% formic acid in 80% acetonitrile (solvent B) via centrifugation for each elution into the same tube.

14. Dry the elutes in a vacuum concentrator.

15. Reconstitute peptides in 10 μL of solvent A for LC–MS/MS.

**C. Mass spectrometry proteomics**

1. Perform LC–MS/MS proteomics as desired. Standard procedures were performed using a Q Exactive HF-X coupled with an UltiMate 3000 RSLCnano system.

a. Load peptides on a C18 column. For chromatographic separation, a flow rate of 300 nL/min was used with the following 120 min gradient: 96% A + 4% Y for 18 min, 70% A + 30% Y for 72 min, 60% A + 40% Y for 15 min, and 4% A + 96% Y for 15 min, where solvent A was 0.1% formic acid in LC–MS-grade water and solvent Y was 0.1% formic acid in LC–MS-grade acetonitrile.

b. Full MS scans were acquired at a resolution of 60,000, with an automatic gain control (AGC) target of 3 × 10^6^, maximum injection time (IT) of 20 ms, and scan range of 300–1,650 m/z in a data-dependent mode. MS2 scans were acquired with the following parameters: resolution of 15,000, AGC target of 1× 10^5^, maximum IT of 54 ms, loop count 15, TopN 15, isolation window 1.4 m/z, fixed first mass 100.0 m/z, normalized collision energy (NCE) 28 units, charge exclusion of unassigned, 1, 6–8, and > 8, peptide match preferred, exclude isotopes on, and fragmented m/z values dynamically excluded from further selection for a period of 45 s.

## Data analysis

1. Process and analyze raw data using appropriate software. Raw data in our experiments were processed and analyzed using MaxQuant and Perseus [14].

2. GO term enrichments were performed using DAVID [15] with the *Mus musculus* proteome as a background (Figure 1C–F). Importantly, GO term analysis identified robust enrichment of proteins localized to the cell membrane and periphery and involved in cell surface processes, including cell junction organization, adhesion, and transport, which are in line with luminal cerebrovascular proteome enrichment.

3. Further analyses looking at differential protein expressions between experimental groups can be conducted using RStudio or equivalent platforms. Please refer to Shi et al. [4] for additional details on data processing and analysis (Figure 1G). Raw mass spectrometry data from this study have been deposited in the PRIDE database under accession code PXD043964.

## Validation of protocol

This protocol (or parts of it) has been used and validated in the following research article(s):

• Shi et al. [4]. Glycocalyx dysregulation promotes blood-brain barrier dysfunction in ageing and disease. *Nature.* (Extended Data Figure 4A–F). Key validations using brain section imaging, western blot, GO term enrichment analysis of identified proteins, detection of known luminal candidates, and differential expression analysis between experimental groups are also depicted in Figure 1.

## General notes and troubleshooting

**General notes**

1. A detailed Bio-protocol for brain microvessel isolation (steps A4–20) with helpful procedural images is described in Buff et al. [11].

2. Following brain extraction (step A3), carry out all subsequent microvessel isolation steps on ice or in a cold room to minimize protein changes and degradation.

3. Use proper protein handling practices to avoid contamination of samples intended for mass spectrometry. This includes working in a clean environment (e.g., a laminar flow hood), wearing clean, powder-free gloves, and avoiding exposure to common environmental contaminants such as dust, skin cells, and hair.

4. While we use mass spectrometry–based proteomics for protein identification, other proteomic platforms, such as antibody- and aptamer-based protein detection methods, may also be used with this protocol if preferred or if mass spectrometry is not available. These methods must be tested for compatibility with NHS-ester labeling of proteins.

5. Although demonstrated here in mice, this method is expected to be broadly applicable to other perfusable organisms for profiling the cerebrovascular proteome.

**Troubleshooting**

Problem 1: Ineffective cerebrovascular biotinylation.

Possible cause: Ineffective perfusion or sulfo-NHS-biotin quenching occurred.

Solution: Make sure to prepare sulfo-NHS-biotin solution fresh and use it immediately. Make sure perfusion successfully clears out blood from the vasculature and that the needle remains securely positioned in the left ventricle throughout the procedure to maintain consistent and efficient perfusion.

Problem 2: Small or no microvessel pellet after step A13.

Possible cause: Brain tissue may have been over-homogenized.

Solution: Make sure the pestle is loosely fit. Make sure chopping does not result in too fine tissue chunks. Try douncing with fewer strokes until the tissue is just homogenized and avoid excessively twisting the pestle.

Problem 3: Low yield of microvessels after step A20.

Possible cause: Microvessels may have been lost during the collection process.

Solution: Be sure to wash all mesh parts of the strainer, including the sides, to collect all microvessels. Additionally, be careful to wash through the strainer mesh without the liquid running off the outside of the strainer. Always inspect the strainer carefully after washing to ensure all microvessels have been collected.

Problem 4: High non-vascular background signal in protein identifications.

Possible cause: Inadequate washing of streptavidin beads, leading to carryover of nonspecifically bound proteins.

Solution: Ensure thorough washing of streptavidin beads with each wash buffer prior to on-bead digestion. Perform all washes with sufficient volume and gentle agitation to minimize background contamination while preserving bead-bound target proteins.
